# Therapeutic hypothermia in adult patients receiving extracorporeal life support: early results of a randomized controlled study

**DOI:** 10.1186/s13019-016-0437-8

**Published:** 2016-04-05

**Authors:** Philip Y.K. Pang, Gillian H.L. Wee, Anne E.E. Hoo, Ismail Mohamed Tahir Sheriff, See Lim Lim, Teing Ee Tan, Yee Jim Loh, Ka Lee Kerk, Yoong Kong Sin, Chong Hee Lim

**Affiliations:** Department of Cardiothoracic Surgery, National Heart Centre Singapore, 5 Hospital Drive, 169609 ᅟ, Singapore; Cardiothoracic Intensive Care Unit, National Heart Centre Singapore, ᅟ, Singapore; Perfusion Unit, National Heart Centre Singapore, ᅟ, Singapore; Mechanical Circulatory Support, Heart and Lung Transplant Unit, National Heart Centre Singapore, ᅟ, Singapore

**Keywords:** Cardiac arrest, Therapeutic hypothermia, Extracorporeal life support

## Abstract

Cardiac arrest with cerebral ischaemia frequently leads to severe neurological impairment. Extracorporeal life support (ECLS) has emerged as a valuable adjunct in resuscitation of cardiac arrest. Despite ECLS, the incidence of permanent neurological injury remains high. We hypothesize that patients receiving ECLS for cardiac arrest treated with therapeutic hypothermia at 34 °C have lower neurological complication rates compared to standard ECLS therapy at normothermia. Early results of this randomized study suggest that therapeutic hypothermia is safe in adult patients receiving ECLS, with similar complication rates as ECLS without hypothermia. Further studies are warranted to measure the efficacy of this therapy.

## Introduction

Cardiac arrest with widespread cerebral ischaemia frequently leads to severe neurological impairment. Extracorporeal life support (ECLS) is a valuable adjunct in resuscitation of cardiac arrest and is instituted whenever indicated [1]. Despite ECLS, the rate of survival to hospital discharge with good neurological function remains low, ranging from 26 to 47 % [2–4].

Ischaemia has a key role in all forms of brain injury and preventing ischaemic (or secondary) injury is at the core of all neuroprotective strategies. Therapeutic induced hypothermia via surface cooling has been shown to lower the rate of neurological complications in patients resuscitated from cardiac arrest by up to 23 % [5, 6].

ECLS is an ideal tool for the institution of cooling as the extracorporeal pump can achieve flow rates of up to 5 L/min. This allows for rapid and homogenous cooling and subsequent rewarming via large bore cannulas placed in the common femoral artery and vein. Cooling via the ECLS circuit can be augmented with surface cooling using cooling blankets and ice packs. To date, there are few reports from studies evaluating the use of therapeutic hypothermia in adult patients receiving ECLS [2–4, 7]. The objective of this study is to evaluate the safety and clinical efficacy of therapeutic induced hypothermia in patients receiving ECLS following cardiopulmonary resuscitation (CPR) for cardiac arrest.

## Methods

Following approval from the SingHealth institutional review board (reference: 2013/153/C), a randomized controlled study was commenced at our tertiary referral center, to investigate the outcome of patients who remain unconscious after initiation of ECLS for cardiac arrest. All patients received good quality CPR and were adequately resuscitated by qualified medical staff prior to commencement of ECLS. Patients in the control group received ECLS at normothermia (37 °C) whereas the treatment group received ECLS at induced hypothermia (34 °C) for 24 h. The target recruitment is 50 patients over a period of 36 months.

## Inclusion criteria

Cardiac arrest patients with ECLS instituted and any of the following:

- Ventricular fibrillation
- Asystole or pulseless electrical activity (PEA)
- Pulseless ventricular tachycardia
- Downtime less than 45 min. Defined as the time from the onset of cardiac arrest to the initiation of Advanced Cardiac Life Support (ACLS)
- Comatose patients
- Patients not responding appropriately to verbal commands after return of spontaneous circulation (ROSC).
- Total ACLS time <60 min
- Age ≥21 years
- Intubated with mechanical ventilation

## Exclusion criteria

- Patients responding appropriately to verbal commands after ROSC
- CPR longer than 45 min
- Severe coagulopathy with clinical evidence of bleeding and/or platelets less than 30 × 10^3^/mm^3^ and/or INR ≥2.5
- Other causes of coma (e.g. drug overdose, head trauma, stroke, overt status epilepticus)
- Positive pregnancy test in women aged below 50 years
- Premorbid status bedbound and uncommunicative
- Temperature <30 °C after cardiac arrest

Eligible patients were randomly assigned to hypothermia or normothermia according to the day of the month, with patients assigned to hypothermia on odd-numbered days. The primary outcome measure was survival to hospital discharge with sufficiently good neurological function to be discharged home or to a rehabilitation facility, defined as a cerebral performance category (CPC) of 1–2. The CPC, shown in Table 1, is a commonly used 5-category scale which has been the historical gold standard for measuring neurological status after cardiac arrest [8, 9]. The 5 categories are: CPC 1, conscious and alert with good cerebral performance; CPC 2, conscious and alert with moderate cerebral performance; CPC 3, conscious with severe cerebral disability; CPC 4, comatose or in persistent vegetative state; and CPC 5, brain dead, circulation preserved. Good and poor neurological outcomes were defined as a CPC of 1–2 and 3–5 respectively. Secondary end points were mortality within 6 months, the length of in-hospital stay and the rate of ECLS-related complications within seven days.

**Table 1:** Cerebral performance category

CPC 1	Conscious, alert, able to work and lead a normal life. May have minor psychological or neurological deficits (mild dysphasia, non-incapacitating hemiparesis, or minor cranial nerve abnormalities).
CPC 2	Conscious. Sufficient cerebral function for part-time work in sheltered environment or independent activities of daily life (dress, travel by public transportation, food preparation). May have hemiplegia, seizures, ataxia, dysarthria, or permanent memory or mental changes.
CPC 3	Conscious. Dependent on others for daily support (in an institution or at home with exceptional family effort). Has at least limited cognition. This category includes a wide range of cerebral abnormalities, from patients who are ambulatory but have severe memory disturbances or dementia precluding independent existence, to those who are paralyzed and can communicate only with their eyes, as in the “locked in” syndrome.
CPC 4	Unconscious. Unaware of surroundings, no cognition. No verbal and/or psychological interaction with environment.
CPC 5	Brain dead, circulation preserved.

*CPC* Cerebral performance category

## ECLS Setup & management

ECLS was instituted in all patients via percutaneous cannulation of the common femoral artery and vein. Standard ECLS components consisting of an extracorporeal centrifugal pump, oxygenator and heat exchanger were utilized. In patients randomized to receive therapeutic induced hypothermia, cooling to a target core body temperature of 34 °C was performed by modulating the heat exchanger component of the ECLS circuit. The temperature was maintained at 34 °C for 24 h in the intensive care unit. Core body temperature readings were obtained by measuring the tympanic and nasopharyngeal temperatures as well as via sensors of the Allon 2000 (Allon, MTRE, Israel) temperature regulating console. An intra-aortic balloon pump was inserted in all patients to augment haemodynamic support. Intravenous heparin was administered to prevent thrombosis within the ECLS circuit, unless contraindicated by existing bleeding.

## Statistical analysis

Statistical analyses were performed with the Statistical Package for Social Science, version 17 (SPSS, Chicago, IL, USA). Continuous variables were expressed as either means with standard deviation or median with interquartile range, as appropriate. These were compared using two-tailed *t*-test or Mann–Whitney *U*-test, respectively. Categorical variables, expressed as percentages, were analyzed with *χ*2 or Fisher’s exact test. All two-tailed *P*-values <0.05 were taken as significant.

## Findings

From Aug 2013 to Jan 2015, 21 patients (9 hypothermia, 12 normothermia) received ECLS as salvage therapy for cardiac arrest. Patient demographics and baseline clinical data are shown in Table 2. The mean age was 52.5 ± 11.0 years. Seventeen patients (81.0 %) were male. Nineteen patients (90.5 %) suffered a witnessed in-hospital cardiac arrest. Seventeen events (81.0 %) were attributable to acute coronary syndrome.

**Table 2:** Patient demographics and baseline clinical data

Variable	All patients *n* = 21 (%)	Hypothermia *n* = 9 (%)	Normothermia *n* = 12 (%)	*P*-value
Demographics				
Age (years)	52.5 ± 11.0	45.9 ± 12.2	57.4 ± 7.0	0.013
Gender (male)	17 (81.0)	8 (88.9)	9 (75.0)	0.422
BSA (m^2^)	1.72 ± 0.18	1.71 ± 0.16	1.72 ± 0.20	0.930
Comorbidities				
Hypertension	11 (52.4)	4 (44.4)	7 (58.3)	0.528
Hyperlipidaemia	10 (47.6)	6 (50.0)	4 (44.4)	0.801
Diabetes mellitus	9 (42.9)	5 (41.7)	4 (44.4)	0.899
Renal failure (CrCl <60 ml/min)	12 (57.1)	5 (55.6)	7 (58.3)	0.899
Coronary artery disease	17 (81.0)	6 (66.7)	11 (91.7)	0.149
Peripheral vascular disease	3 (14.3)	1 (11.1)	2 (16.7)	0.719
Previous stroke	1 (4.8)	0 (0)	1 (8.3)	0.375
Atrial fibrillation	1 (4.8)	0 (0)	1 (8.3)	0.375
Smoking	12 (57.1)	6 (66.7)	6 (50.0)	0.445
LVEF (%)	23.4 ± 15.8	13.9 ± 3.6	30.3 ± 17.8	0.013

Values for parametric continuous variables are expressed as mean ± standard deviation. Values for categorical variables are expressed as numbers (%)

*BSA* body surface area, *CrCl* creatinine clearance, *LVEF* left ventricular ejection fraction

Pre-ECLS data are shown in Table 3. The initial rhythm was pulseless ventricular tachycardia or ventricular fibrillation in 7 patients (33.3 %), pulseless electrical activity in 10 (47.6 %) and asystole in 4 (19.0 %). The mean duration of CPR and ECLS were respectively, 25.7 min and 4.4 days. Target cooling to 34 °C was achieved in all 9 patients assigned to the hypothermia arm. ECLS-related complications are shown in Table 4. There were no intergroup differences in the frequency of ECLS-related adverse events.

**Table 3:** Pre-ECLS data

Variable	All patients *n* = 21 (%)	Hypothermia *n* = 9 (%)	Normothermia *n* = 12 (%)	*P*-value
Initial rhythm				
Pulseless VT/VF	7 (33.3)	4 (44.4)	3 (25.0)	0.350
PEA	10 (47.6)	4 (44.4)	6 (50.0)	0.801
Asystole	4 (19.0)	1 (11.1)	3 (25.0)	0.422
In-hospital cardiac arrest	19 (90.5)	7 (77.8)	12 (100)	0.086
Duration of CPR (mins)	25.7 ± 15.6	29.9 ± 15.6	22.6 ± 15.5	0.300

Values for parametric continuous variables are expressed as mean ± standard deviation. Values for categorical variables are expressed as numbers (%)

*CPR* cardiopulmonary resuscitation, *PEA* pulseless electrical activity, *VF* ventricular fibrillation, *VT* ventricular tachycardia

**Table 4:** ECLS-related complications

Variable	All patients *n* = 21 (%)	Hypothermia *n* = 9 (%)	Normothermia *n* = 12 (%)	*P*-value
Duration of ECLS (days)	4.4 ± 2.7	4.1 ± 1.8	4.6 ± 3.2	0.675
Bleeding	5 (23.8)	2 (22.2)	3 (25.0)	0.882
Intracranial haemorrhage	1 (4.8)	0 (0)	1 (8.3)	0.375
Intrathoracic or intra-abdominal	3 (14.3)	1 (11.1)	2 (16.7)	0.719
PCT / day of ECLS (units)	2.6 ± 2.0	2.2 ± 1.7	2.9 ± 2.2	0.484
New arrhythmias	4 (19.0)	2 (22.2)	2 (16.7)	0.748
Limb ischaemia	6 (28.6)	2 (22.2)	4 (33.3)	0.577
Distal perfusion cannula	13 (61.9)	6 (66.7)	7 (58.3)	0.697
Acute renal failure	19 (90.5)	8 (88.9)	11 (91.7)	0.830
CRRT	18 (85.7)	7 (77.8)	11 (91.7)	0.368
Pneumonia	7 (33.3)	1 (11.1)	6 (50.0)	0.061

Values for parametric continuous variables are expressed as mean ± standard deviation. Values for categorical variables are expressed as numbers (%)

*CRRT* continuous renal replacement therapy, *ECLS* Extracorporeal life support, *PCT* packed cell transfusion

Follow-up data are shown in Table 5. Twelve patients (57.1 %) were successfully weaned off ECLS, of which 5 (23.8 %) survived to hospital discharge and were alive at 6 months follow-up. The causes of death in the 15 patients who died were multi-system organ failure in 12 patients (80.0 %) and severe anoxic brain injury in 3 patients (20.0 %). The median follow-up period for in-hospital survivors was 191 (85, 399) days. Two patients (22.2 %) in the hypothermia group, compared to 1 (8.3 %) in the normothermia group, survived with a good neurological outcome.

**Table 5:** Follow-up data

Variable	All patients *n* = 21 (%)	Hypothermia *n* = 9 (%)	Normothermia *n* = 12 (%)	*P*-value
Weaned from ECLS	12 (57.1)	4 (44.4)	8 (66.7)	0.309
Severe neurological dysfunction	13 (61.9)	6 (66.7)	7 (58.3)	0.697
Length of hospital stay (days)	7 (2, 22)	6 (2, 23)	7 (2, 22)	0.772
In-hospital death	15 (71.4)	6 (66.7)	9 (75.0)	0.676
Hospital discharge	5 (23.8)	3 (33.3)	2 (18.2)	0.436
Survival at 6 months	5 (23.8)	3 (33.3)	2 (18.2)	0.436
Survival (good neurological function)	3 (14.3)	2 (22.2)	1 (8.3)	0.368

Values for non-parametric continuous variables are expressed as median followed by 25th and 75th percentiles. Values for categorical variables are expressed as numbers (%)

*ECLS* extracorporeal life support

## Discussion

A recent large randomized trial has shown a lack of benefit of hypothermia at a targeted temperature of 33 °C compared with a targeted temperature of 36 °C in terms of survival and preservation of cognitive function in unconscious survivors of out-of-hospital cardiac arrest [10, 11]. Despite these findings, therapeutic hypothermia may still benefit patients suffering from refractory cardiac arrest with prolonged resuscitation unresponsive to conventional CPR, who require ECLS as salvage therapy. ECLS-assisted CPR has been reported to improve survival and neurological outcomes compared to conventional CPR. Overall survival and survival with good neurological function rates range from 31 to 50 and 26 to 47 % respectively [2–4, 12].

Survival to hospital discharge and survival with good neurological function in patients receiving therapeutic hypothermia in our randomized cohort were 33.3 and 22.2 % respectively. Superior results have been reported from the recent CHEER trial, during which therapeutic hypothermia (33 °C maintained for 24 h) in conjunction with ECLS was instituted for 24 patients, of which 13 patients (54 %) were successfully weaned from ECMO support. Survival to hospital discharge with full neurological recovery (CPC score 1) occurred in 12 patients (50 %) [7].

The main limitation of this study is the small number of surviving patients and total number of patients recruited to date, thus limiting statistical power. Further study recruitment may shed more light on the benefit of therapeutic hypothermia in adult patients receiving ECLS. In addition, patients receiving hypothermia were significantly younger, with possibly greater potential for neurological recovery compared to older patients.

## Conclusion

The preliminary results of this randomized study suggest that therapeutic hypothermia is safe to use in adult patients receiving ECLS, with similar complication rates compared with ECLS without hypothermia. Further studies are warranted to measure the efficacy of this therapy.

## Abbreviations

ACLS: advanced cardiac life support BSA body surface area
CPC: cerebral performance category
CPR: cardiopulmonary resuscitation
CrCl: creatinine clearance
CRRT: continuous renal replacement therapy ECLS extracorporeal life support LVEF left ventricular ejection fraction​
PCT: packed cell transfusion
PEA: pulseless electrical activity
ROSC: return of spontaneous circulation
VF: ventricular fibrillation
VT: ventricular tachycardia
